# Gout and subsequent erectile dysfunction: a population-based cohort study from England

**DOI:** 10.1186/s13075-017-1322-0

**Published:** 2017-06-06

**Authors:** Alyshah Abdul Sultan, Christian Mallen, Richard Hayward, Sara Muller, Rebecca Whittle, Matthew Hotston, Edward Roddy

**Affiliations:** 1 Arthritis Research UK Primary Care Centre, Research Institute for Primary Care & Health Sciences, Keele University, Keele, Staffordshire, ST5 5BG UK; 2 Royal Cornwall Hospital, Treliske, Truro, Cornwall TR1 3LQ UK

**Keywords:** Gout, Erectile dysfunction, Incidence rate, Epidemiology

## Abstract

**Background:**

An association has been suggested between gout and erectile dysfunction (ED), however studies quantifying the risk of ED amongst gout patients are lacking. We aimed to precisely determine the population-level absolute and relative rate of ED reporting among men with gout over a decade in England.

**Methods:**

We utilised the UK-based Clinical Practice Research Datalink to identify 9653 men with incident gout age- and practice-matched to 38,218 controls. Absolute and relative rates of incident ED were calculated using Cox regression models. Absolute rates within specific time periods before and after gout diagnosis were compared to control using a Poisson regression model.

**Results:**

Overall, the absolute rate of ED post-gout diagnosis was 193 (95% confidence interval (CI): 184–202) per 10,000 person-years. This corresponded to a 31% (hazard ratio (HR): 1.31 95%CI: 1.24–1.40) increased relative risk and 0.6% excess absolute risk compared to those without gout. We did not observe statistically significant differences in the risk of ED among those prescribed ULT within 1 and 3 years after gout diagnosis. Compared to those unexposed, the risk of ED was also high in the year before gout diagnosis (relative rate = 1.63 95%CI 1.27–2.08). Similar findings were also observed for severe ED warranting pharmacological intervention.

**Conclusions:**

We have shown a statistically significant increased risk of ED among men with gout. Our findings will have important implications in planning a multidisciplinary approach to managing patients with gout.

## Background

Gout is the most prevalent type of inflammatory arthritis, affecting 2.4% of adults in the UK. It is largely managed in primary care [1] and is associated with a number of comorbidities [2, 3]. Erectile dysfunction (ED) is also a common problem in the general population affecting 2% of men aged under 40 years rising to 86% of those aged over 80 years [4]. Recently, an association between gout and ED had been suggested based on one small hospital-based cross-sectional study [5] and two cohort studies from Southeast Asia [6, 7]. Whilst the latter studies were based on large administrative data, their findings can only be generalised to western countries with caution, given the large variation in the reporting of ED by region [4]. Furthermore, the causal relationship between gout and ED demonstrated in one previous study failed to account for important factors including body mass index (BMI), smoking status and alcohol consumption, which may have confounded the association [6]. Finally, to our knowledge no previous study has quantified the incidence of ED warranting pharmacological intervention or assessed ED reporting both before and after gout diagnosis, which may be important in understanding disease mechanisms. Knowledge about gout-associated comorbidities is crucial for planning multidisciplinary approaches to disease management. Therefore, the aim of this study was to precisely determine the population level absolute and relative rates of ED reporting among men with gout over a decade in the UK.

## Methods

The Clinical Practice Research Datalink (CPRD) [8] is a large longitudinal UK database that contains computerised primary care (i.e. general practice) records of anonymised patients. Approximately 98% of the England and Wales population are registered with general practitioners (GPs), who are responsible for almost the entirety of a patient’s medical care, including coordination of their health care from a hospital or other secondary care facilities. The CPRD includes practices that have received training to record information using Vision software and that have consented to be included in the database. All patients within a consented practice are automatically included. CPRD is subjected to various quality checks and a practice’s data is only used when it is of the highest quality to be used for research. This is denoted by defining an up-to-standard (UTS) time period for each practice. For the purpose of this study, we utilised a subset of CPRD linked to Hospital Episode Statistics, which records England’s secondary care data. The HES-linked CPRD have been compared to the Office of National Statistics (ONS) data showing similar age and sex distribution [9].

We identified men aged 18–64 years with a first-ever recorded diagnosis of gout from general practice between 1998 and 2004 who were then followed up until 2015. The study start date was defined as the latest of study start date (1 January 1998), date of patient registration with the practice and UTS date. The study end date was defined as the earliest of the date of last data collection from the practice, date patient transferred out from practice or died, and the study end date (31 August 2015). Gout diagnosis was based on a medical code assigned by the physician which has been previously validated in CPRD with a positive predictive value of 90% [10]. Each patient was assigned an index date corresponding to the date of their gout diagnosis and randomly matched to four controls on age (±3 years) without gout or a prescription for urate-lowing therapy (ULT) who were registered at the same practice, and were alive and contributing data at the index date. Controls were assigned the same index date as their matched gout case. Those with less than a year of follow-up, history of prior ED diagnosis, or prescribed ED-specific medication in the absence of a diagnostic code during the study period were excluded. For our primary analysis, we looked at the rate of ED reporting after the index date (for cases and controls). In order to assess how ED reporting rates varied in relation to the time of gout diagnosis, we also analysed all available person-time before the index date. Thus we were able to look at patients who developed ED before their gout diagnosis and subsequently excluded from our primary analysis.

Incident ED reporting was based on medical codes assigned by the physician during the study period. In order to ensure incident reporting, those assigned an ED code in the first 6 months of their registration with the practice were classified as prevalent cases and were excluded from our post-gout analysis. We only assessed the incidence of the first ED and therefore any subsequent ED events (along with the follow-up time) were excluded from the study. We defined pharmacologically treated ED based on those who were prescribed pharmacological treatment for ED any time after their initial diagnosis.

We extracted information on lifestyle-related characteristics (BMI, smoking status and alcohol consumption) and specific comorbidities (ischemic heart disease, hypertension, diabetes mellitus, depression and chronic renal disease) for both cases and controls. BMI was categorised as normal weight (18.5–25 kg/m^2^), underweight (≤18.5 kg/m^2^), overweight (>25– 29.9 kg/m^2^) and obese (≥30 kg/m^2^). Covariate information was ascertained using the most recent measure before the outcome or study end date.

Patient characteristics were compared between cases and controls using frequencies and percentages. Chi-squared tests were conducted to quantify statistically significant differences between cases and controls. We calculated the incidence of ED as the number of first-recorded ED diagnoses per 10,000 person-years. Adjusted hazard ratio (HR) and 95% confidence interval (CI) was calculated using a Cox regression model to compare the hazard rate between cases and controls. Robust standard errors were used to account for matching. To explore potential interaction by lifestyle-related characteristics and comorbidities, we stratified our analyses by those covariates. Those with missing information on BMI were categorised as a separate category and were included in the analysis. We assessed the timing of ED reporting in relation to gout diagnosis by comparing the absolute rate of ED before and after gout diagnosis among cases and controls in terms of incidence rate ratios using a Poisson regression model. We utilised landmark analysis to examine the effect of ULT on the risk of ED [11]. This method deals with immortal time bias which biases the results in favour of the treatment under study by granting a spurious survival advantage to the treated group. In landmark analysis [12], a fixed time after the initiation of therapy is selected a priori for conducting survival analysis. Only patients alive and contributing data at landmark time were included in the analysis. The exposure was evaluated between the index date and the landmark time whereas the incidence of ED was only considered after the landmark time point. Two landmark points were considered in the analysis (1 and 3 years after diagnosis) based on a previously published study [13]. Only patients prescribed more than 6 months of ULT were considered to be exposed. Finally, we repeated our analyses for pharmacologically treated ED. All statistical analyses were carried out using STATA MP 14 (StataCorp LP, College Station, TX, USA).

## Results

Our gout cohort consisted of 9653 patients matched to 38,218 controls representing 90,036 and 302,814 person-years of follow-up, respectively. The median follow-up from index to study end date was calculated to be 10 years (IQR = 5–13 years). Compared to controls, men with gout were less likely to smoke (15% versus 22%; *p* < 0.001), more likely to drink ≥ 10 alcohol units/week (49% versus 32%, *p* value < 0.001), and more likely to be overweight or obese (Table 1). Gout cases also had a higher prevalence of diabetes mellitus, hypertension, ischemic heart disease, chronic renal disease and depression compared to their matched controls.

**Table 1:** Basic characteristics of the study population

Variable	Controls *N* = 38,218		Gout cases *N* = 9653		*p* value*
	N	%	N	%	
Age in years					
≤ 34	2774	7.3	712	7.4	0.800
35–44	8900	23.3	2206	22.9	
45–54	12,887	33.7	3256	33.7	
55–64	13,649	35.7	3479	36.0	
Never/ex-smokers	30,010	78.5	8210	85.1	<0.001
Current smoker	8200	21.5	1443	14.9	
Never/ex-drinkers	10,703	28.0	1578	16.3	<0.001
Current drinkers (1–9 units/week)	15,420	40.4	3381	35.0	
Current drinkers (≥10 units/week)	12,087	31.6	4694	48.6	
Body mass index					
Normal	10203	26.7	1376	14.3	<0.001
Underweight	332	0.9	42	0.4	
Overweight	13,224	34.6	3753	38.9	
Obese	6952	18.2	3598	37.3	
Missing	7499	19.6	884	9.2	
Comorbidities					
Ischemic heart disease	2893	7.6	1180	12.2	<0.001
Diabetes	3084	8.1	1470	15.2	<0.001
Hypertension	8210	21.5	4191	43.4	<0.001
Chronic renal disease	1026	2.7	802	8.3	<0.001
Depression	6242	16.3	1894	19.6	<0.001

*Chi-squared test

A total of 5860 incident ED events were recorded during the study period after the index date (Table 2). The absolute rate (AR) of ED reporting among men with gout was calculated to be 193 (95%CI; 184–202) per 10,000 person-years. This corresponded to a 0.6% excess absolute risk and a 31% increased adjusted relative risk (HR = 1.31; 95%CI 1.24–1.40) compared to those without gout. Our results remained consistent when we stratified our analyses by patient characteristics. The absolute rate of pharmacologically treated ED among men with gout was 107 per 10,000 person years (95%CI 107–121) with the excess absolute risk of 0.3% compared with controls (Table 3). However, the risk of pharmacologically treated ED was not statistically different between cases and controls among younger population (<45 years) or those with a recorded diagnosis of diabetes mellitus or chronic renal failure.

**Table 2:** Absolute and relative rate of ED reporting by patient characteristics and comorbidities

Variable	Controls				Gout cases				Adjusted^a^ HR	95% CI		Absolute risk difference^b^
	N	Rate^b^	95% CI		N	Rate^b^	95% CI					
Overall	4124	136.2	132.1	140.4	1736	192.8	184.0	202.1	1.31	1.24	1.40	56.6
Age												
≤ 34	68	34.6	27.3	43.9	42	61.4	45.4	83.1	1.20	0.79	1.83	26.8
35–44	548	76.2	70.1	82.8	251	112.6	99.5	127.4	1.18	1.01	1.39	36.4
45–54	1551	146.0	138.9	153.5	653	211.7	196.1	228.6	1.28	1.16	1.41	65.7
55–64	1957	186.3	178.3	194.8	790	262.8	245.1	281.8	1.37	1.25	1.49	76.5
Smoking status												
Never/ex-smokers	3281	132.8	128.4	137.5	1487	191.2	181.7	201.1	1.32	1.24	1.41	58.3
Current smoker	843	151.1	141.2	161.6	249	203.2	179.5	230.1	1.29	1.11	1.50	52.2
Alcohol consumption												
Never/ex-drinkers	834	112.3	104.9	120.2	283	206.7	183.9	232.2	1.77	1.53	2.05	94.4
Current drinkers (1–9 units/week)	1716	132.6	126.5	139.1	601	184.2	170.1	199.5	1.29	1.17	1.42	51.6
Current drinkers (≥10 units/week)	1574	158.7	151.1	166.8	852	194.9	182.2	208.4	1.21	1.11	1.32	36.2
Body mass index												
Normal	943	117.4	110.1	125.1	201	157.8	137.4	181.1	1.34	1.14	1.57	40.4
Underweight	18	67.5	42.5	107.2	-	-	-	-	-	-	-	-
Overweight	1659	146.5	139.6	153.7	696	196.5	182.4	211.6	1.33	1.21	1.45	50.0
Obese	1068	170.0	160.1	180.5	712	204.2	189.7	219.7	1.23	1.11	1.35	34.1
Missing	436	99.6	90.7	109.4	126	189.5	159.1	225.6	1.71	1.37	2.14	89.8
Comorbidities												
Congestive heart disease	443	174.2	158.7	191.2	253	227.6	201.2	257.5	1.26	1.07	1.47	53.4
Diabetes	946	343.6	322.4	366.2	514	349.4	320.4	380.9	1.05	0.94	1.74	5.8
Hypertension	1347	173.4	164.4	183.0	852	206.8	193.4	221.2	1.18	1.09	1.29	33.4
Chronic renal disease	122	106.3	89.0	127.0	129	149.5	125.8	177.7	1.41	1.10	1.81	43.2
Depression	872	168.5	157.7	180.0	381	216.1	195.4	238.9	1.26	1.11	1.43	47.6

*ED* erectile dysfunction, *CI* confidence interval

^a^Mutually adjusted unless stratified

^b^Per 10,000 person-years

**Table 3:** Absolute and relative rate of pharmacologically treated ED by patient characteristics and comorbidities

Variable	Controls				Gout cases				Adjusted^a^ HR	95% CI		Absolute risk difference^b^
	N	Rate^b^	95% CI		N	Rate^b^	95% CI					
Overall	2429	80.2	77.1	83.5	1024	113.7	107.0	120.9	1.36	1.26	1.47	33.5
Age												
≤ 34	32	16.3	11.5	23.0	23	33.6	22.4	50.6	1.24	0.67	2.29	17.3
35–44	329	45.7	41.1	51.0	139	62.4	52.8	73.6	1.11	0.89	1.37	16.6
45–54	925	87.1	81.6	92.9	391	126.8	114.8	140.0	1.30	1.15	1.47	39.7
55–64	1143	108.8	102.7	115.3	471	156.7	143.2	171.5	1.46	1.30	1.64	47.9
Smoking status												
Never/ex-smokers	1955	79.1	75.7	82.7	891	114.5	107.3	122.3	1.39	1.27	1.51	35.4
Current smoker	474	84.9	77.6	92.9	133	108.5	91.6	128.7	1.23	1.01	1.51	23.6
Alcohol consumption												
Never/ex-drinkers	484	65.2	59.6	71.2	154	112.5	96.0	131.7	1.82	1.49	2.21	47.3
Current drinkers (1–9 units/week)	992	76.7	72.0	81.6	321	98.4	88.2	109.8	1.27	1.11	1.44	21.7
Current drinkers (≥10 units/week)	953	96.1	90.2	102.4	549	125.6	115.5	136.5	1.31	1.17	1.46	29.5
Body mass index												
Normal	589	73.3	67.6	79.5	116	91.0	75.9	109.2	1.27	1.03	1.56	17.7
Underweight	10	37.5	20.2	69.7		-	-	-	-	-	-	-
Overweight	971	85.8	80.5	91.3	419	118.3	107.5	130.2	1.36	1.21	1.53	32.5
Obese	581	92.5	85.3	100.3	399	114.4	103.7	126.2	1.28	1.12	1.46	21.9
Missing	278	63.5	56.5	71.4	89	133.8	108.7	164.7	1.88	1.43	2.48	70.3
Comorbidities												
Congestive heart disease	199	78.2	68.1	89.9	123	110.7	92.7	132.1	1.34	1.06	1.70	32.4
Diabetes	488	177.3	162.2	193.7	264	179.4	159.1	202.4	1.04	0.89	1.21	2.2
Hypertension	737	94.9	88.3	102.0	486	118.0	107.9	128.9	1.24	1.10	1.39	23.1
Chronic renal disease	51	44.4	33.8	58.5	52	60.3	45.9	79.1	1.34	0.90	1.99	15.8
Depression	479	92.6	84.6	101.2	219	124.2	108.8	141.8	1.37	1.16	1.62	31.6

*ED* erectile dysfunction, *HR* hazard ratio, *CI* confidence interval

^a^Mutually adjusted unless stratified

^b^Per 10,000 person-years

**Fig. 1:**
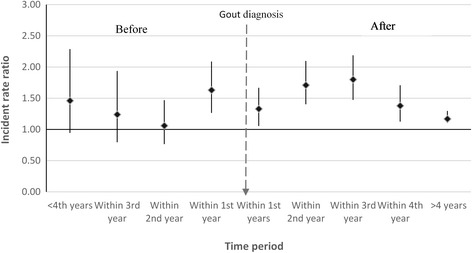
Timing of ED reporting in relation to gout diagnosis compared to controls

**Fig. 2:**
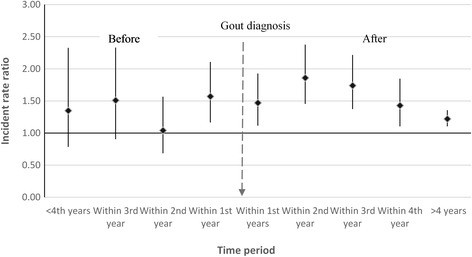
Timing of pharmacologically treated ED reporting in relation to gout diagnosis compared to controls

As shown in Table 4, 1616 patients in the 1-year landmark analysis and 1091 patients in the 3-year landmark analysis reported ED during the follow-up period. We did not find statistically significant differences in the risk of ED by ULT. Similar findings were also reported for pharmacologically treated ED. Those with gout had 1.77 times (95%CI 1.38–2.26) higher relative risk of reporting ED compared to controls within the second year of gout diagnosis. The adjusted relative risk decreased in the years following diagnosis to 1.16 after 5 years of diagnosis but was also 1.63 times higher (95%CI 1.24-2.08) within the year preceding diagnosis (Fig. 1). These estimates remained broadly similar for pharmacologically treated ED (Fig. 2).

**Table 4:** Absolute and relative rate of ED among those ever prescribed urate-lowering therapy (ULT)

	1-year landmark analysis Total patients = 9322 ULT exposed = 1249			3-year landmark analysis Total patients = 8650 ULT exposed = 1787		
Gout cases	ED events	Absolute rate^b^ (95% CI)	Adjusted HR (95% CI)^a^	ED events	Absolute rate^b^ (95% CI)	Adjusted HR (95% CI)^a^
For overall ED reporting						
Not treated	1364	194.8 (184.7–205.4)	1.00	797	196.9 (184.9–209.6)	1.00
Treated with ULT	252	237.1 (209.6–268.3)	1.14 (0.99–1.30)	294	229.1 (204.4–256.9)	1.07 (0.94–1.22)
Pharmacologically treated ED						
Not treated	797	113.8 (106.2–122.0)	1.00	548	110.3 (101.5–120)	1.00
Treated with ULT	141	132.8 (112.5–156.5)	1.12 (0.93–1.34)	161	125.5 (107.5–146.4)	1.08 (0.90–1.29)

*ED* erectile dysfunction, *CI* confidence interval, *HR* hazard ratio

^a^Adjusted for age, BMI, smoking status, alcohol consumption, ischemic heart disease, chronic renal disease, depression, hypertension and diabetes

^b^Per 10,000 person-years

## Discussion

In this large nationally representative cohort of men with gout with more than 10 years of follow-up, we have provided contemporary, generalisable and population-based estimates of the absolute risk of ED reporting in England. Overall we found the risk of ED post-diagnosis to be 2% per year. Whilst those with gout were 31% more likely to consult their general practitioner for ED compared to those without gout, the excess absolute risk was 0.6% per year. The risk of pharmacologically treated ED was not statistically different between cases and controls among the younger population (<45 years) or those with a recorded diagnosis of diabetes or chronic renal failure. Having more than 6 months of ULT within 1 and 3 years from initial gout diagnosis had no impact ED reporting. The risk of consulting a GP for ED was also higher within the year preceding the initial gout diagnosis.

We have conducted one of the largest studies to precisely determine the risk of ED among men with gout. Our study used an open cohort approach, with prospectively recorded data utilising information from primary care from across England, covering 3% of the total UK population with similar age and sex distribution to the population as a whole. Thus these findings are not only generalisable to England but also to other developed nations with comparable health care systems. Our use of primary care data allowed us to adjust our estimates for important confounding factors such as smoking status, alcohol consumption and BMI, which had not been done in previous studies on the subject. Furthermore, prospective data allowed us to look at the timing of ED in relation to gout diagnosis, which also has not been previously demonstrated.

A potential limitation is the use of anonymised data with no direct link to the patient record and our reliance on general practitioners entering data accurately. However, gout diagnosis has been externally validated in CPRD with a high degree of accuracy [10] so it is unlikely that there is any major error in our study due to the misclassification of gout diagnosis. Similarly, validity and under-reporting of ED due to the reluctance of patients to seek medical advice may be a concern, however, our study provides estimates which reflect contemporary ED reporting and screening practices in primary care, which form the basis of management. Nonetheless, our absolute rates of ED are much higher compared to most studies on the subject [6, 7].

There may be potential ascertainment bias as gout patients may be more aware of associated comorbidities and consequently, report more ED compared to those without gout. However, we believe the impact of this may be minimal as higher relative risk of ED was also observed among those with pharmacologically treated ED and also within the year preceding gout diagnosis. We also acknowledge the lack of complete data on BMI which could bias our estimates. Nonetheless, we treated patients with missing data as a separate category and included them in our analysis. The fact that we were able to adjust for BMI and other lifestyle-related characteristics is an advantage over other large studies on the topic. Finally, the use of 1- and 3-year landmarks for our ULT analysis means that our findings are only generalisable to those alive and contributing data at those landmark points and prescribed at least 6 months of ULT after their initial gout diagnosis.

The absolute rate of ED reporting among gout patients in our cohort was calculated to be 2% per year. This is much higher than the rate previously reported by two large registry-based studies from Taiwan (0.1–0.2% per year) utilising Health Insurance Research Database [6, 7]. This may be because treatment for ED in Taiwan is not covered by their National Health Insurance program leading to under-ascertainment of ED. Furthermore, differences in study population and cultural norms may also have contributed to ED under-reporting. Overall we observed a 31% increased relative risk of ED among gout patients compared to controls. Whilst these estimates are in line with those reported in Southeast Asian studies [6, 7], a US-based study reported around threefold higher risk of ED among gout cases [5]. The latter study was based on a cross-sectional survey of men aged 18–89 years attending a rheumatology clinic with any form of arthritis, which may be prone to selection and recall bias. Furthermore, the finding of this previous study may not be generalisable to the majority of patients with gout who are managed exclusively in primary care.

Whilst our study supports most of the findings from the Taiwanese studies, it is important to note that the excess absolute risk of ED among gout patients compared to controls is less than 0.6%. Furthermore, we reported no increased risk of ED reporting among those with diabetes and chronic renal disease, which suggests stronger influence of those conditions on ED than gout. Our findings support the likely physiological influence of hyperuricaemia on vasculature including induction of vascular smooth muscle proliferation, oxidative stress, and activation of the renin-angiotensin axis in vascular beds [14] which begins in asymptomatic hyperuricaemia before the clinical diagnosis of gout.

## Conclusions

Our study has important clinical implications. First, we have found that men with gout are at a higher risk of ED compared to the general population. The increased relative risk is broadly similar when stratified by comorbidities suggesting limited interaction with those factors. These findings may have important implications in planning a multidisciplinary approach to managing patients with gout. Second, we observed increased relative risk of ED within the year before gout diagnosis. Therefore it may be more reasonable to suspect hyperuricaemia as one of the underlying cause of ED among men with asymptomatic hyperuricaemia but are yet to develop clinically apparent gout.

## Abbreviations

AR: Absolute rate
CI: Confidence interval
CPRD: Clinical Practice Research Datalink
ED: Erectile dysfunction
GP: General practitioner
HR: Hazard ratio
ULT: Urate-lowering therapy
